# Clinical and Prognostic Effect of Plasma Fibrinogen in Renal Cell Carcinoma: A Meta-Analysis

**DOI:** 10.1155/2017/9591506

**Published:** 2017-01-05

**Authors:** Yuejun Tian, Mei Hong, Suoshi Jing, Xingchen Liu, Hanzhang Wang, Xinping Wang, Dharam Kaushik, Ronald Rodriguez, Zhiping Wang

**Affiliations:** ^1^Institute of Urology, Lanzhou University Second Hospital, Key Laboratory of Gansu Province for Urological Diseases, Gansu Nephro-Urological Clinical Center, Lanzhou 730030, China; ^2^Drug Discovery Center, School of Chemical Biology and Biotechnology, Peking University Shenzhen Graduate School, Shenzhen 518055, China; ^3^Department of Urology, University of Texas Health Science Center at San Antonio, 7703 Floyd Curl Drive, San Antonio, TX 78229-3900, USA

## Abstract

*Background*. Although numerous studies have shown that plasma fibrinogen is linked to renal cell carcinoma (RCC) risk, the consistency and magnitude of the effect of plasma fibrinogen are unclear. The aim of the study was to explore the association between plasma fibrinogen and RCC prognosis.* Methods*. An electronic search of Embase, PubMed/MEDLINE, and the Cochrane databases was performed to identify relevant studies published prior to June 1, 2016.* Results*. A total of 3744 patients with RCC from 7 published studies were included in the meta-analysis. The prognostic and clinical relevance of plasma fibrinogen are evaluated in RCC patients. Statistical significance of the combined hazard ratio (HR) was detected for overall survival, cancer-specific survival, and disease-free survival. Our pooled results showed that elevated plasma fibrinogen was significantly associated with clinical stage and Fuhrman grading. The level of plasma fibrinogen was not found to be associated with tumor type and gender.* Conclusions*. Elevated plasma fibrinogen is a strong indicator of poorer prognosis of patients with RCC, whereas the plasma fibrinogen is not significantly associated with tumor type. Therefore, plasma fibrinogen could be used in patients with RCC for risk stratification and decision providing a proper therapeutic strategy.

## 1. Introduction

Renal cell carcinoma (RCC) is the third most frequent malignancy in the urogenital system, which represents about 2% to 3% of cancers in adults [1]. Although the diagnosis and therapeutic modalities of RCC have changed remarkably rapidly, up to one-third of patients present with locally advanced or metastatic disease at initial diagnosis, and the subsequent 5-year survival rate of metastatic RCC is only 10% [2–4]. Therefore, prognostic predictors of high-risk RCC are urgently needed.

Plasma fibrinogen, as an acute phase glycoprotein that is commonly associated with the maintenance of hemostasis, has a critical role in both inflammatory responses and cancer progression. A number of studies have shown that plasma fibrinogen level is upregulated in various cancers and may account for progression and metastasis [5–8]. However, there are conflicting findings on the role of plasma fibrinogen and survival outcomes in RCC. For example, Xiao et al. [9] found that plasma fibrinogen level is an effective tumor marker to evaluate lymph node status, clinical stage, and distant metastases. Sasaki and Onishi [10] also demonstrated that plasma fibrinogen was a prognostic factor predicting worse overall survival (OS) in RCC patients. However, Erdem et al. [11] suggested that preexisting plasma fibrinogen had no significant effect on the outcome of localized RCC.

The aim of our overarching systematic review was to provide a comprehensive and up-to-date summary for the role of fibrinogen in RCC. In addition, we completed meta-analyses to quantify the changes in OS, cancer-specific survival (CSS), and disease-free survival (DFS).

## 2. Materials and Methods 

### 2.1. Search Strategy

This meta-analysis was conducted in accordance with the guideline of Preferred Reporting Items for Systematic Reviews and Meta-Analyses [12]. Because the studies included in this meta-analysis have been published, thus no ethical approval is required. A literature search for published original articles was conducted in Embase, PubMed/MEDLINE, and Cochrane databases. The last updated search was carried out on June 1, 2016. The key search items consist of plasma fibrinogen (“fibrinogen” OR “plasma fibrinogen”), renal cell carcinoma (“renal cell cancer” OR “kidney cancer” OR “renal tumor” OR “renal cell carcinoma”), and “prognosis or prognostic or survival or outcome” and relevant variants of these search terms. The search was confined to articles that were published in English. In addition, references of relevant articles were manually searched for potential eligible trials.

### 2.2. Selection Criteria and Definition

The eligible studies were included only if they met the following criteria: (1) articles were published in English; (2) any clinical study comprising the evaluation of plasma fibrinogen on renal cell cancer prognosis was eligible; (3) the authors must offer the hazard ratios (HRs) and their *p* values, or the information that allowed manual calculation of 95% CI in the papers. Accordingly, studies with the following criteria were excluded: (1) reviews and nonoriginal articles; (2) studies not related to RCC; (3) studies that did not analyze the plasma fibrinogen and the clinical features and survival outcome; (4) studies lacking sufficient data to acquire HR and its standard error (SE). When duplicate articles emerged, the one with the largest data set was adopted. Two researchers (MH and SSJ) screened titles and abstracts of all the searched literatures and verified the studies that met the inclusion criteria for next analysis.

### 2.3. Data Extraction and Study Quality

The following information was retrieved independently by 2 reviewers (MH and SSJ) from the final set of literatures: publication year, name of the first author, number of patients enrolled, recruitment period, age of patients, gender ratio, cut-off value, follow-up time, adjusted factors, and Newcastle-Ottawa Scale (NOS) score. The data were extracted from the original articles. If a study provided the results of both multivariate outcome and univariate outcome, we chose the former. There are no standard quality assessment tools for prognostic studies in systematic reviews. Study quality was independently applied according to the “NOS score” for a cohort study that includes 3 domains with 8 items. Studies with scores of 6 or higher were graded as high quality [13].

### 2.4. Statistical Analysis

The pooled HR and its corresponding 95% CI were calculated to assess the association between plasma fibrinogen and patient survival. The pooled OR and its corresponding 95% CI were used to quantitatively determine the association between plasma fibrinogen and the clinical parameters of RCC. Statistical heterogeneity among studies was assessed using Cochran's *Q* test and Higgins *I* ^2^ statistic [14]. A fixed-effect model (Mantel–Haenszel method) was used to calculate parameters when no obvious heterogeneity existed among studies (*I* ^2^ > 50% suggested high heterogeneity). Sensitivity analysis was performed to test the reliability of the total pooled results by sequential omission of individual studies. Publication bias was assessed using funnel plots and Egger's test. All statistical manipulations in this meta-analysis were undertaken using Stata 14.0 software (Stata Corporation, College Station, TX) with 2-tailed *p* values. A *p* value of <0.05 was considered the significance level.

## 3. Results

### 3.1. Study Characteristics

**Figure 1:**
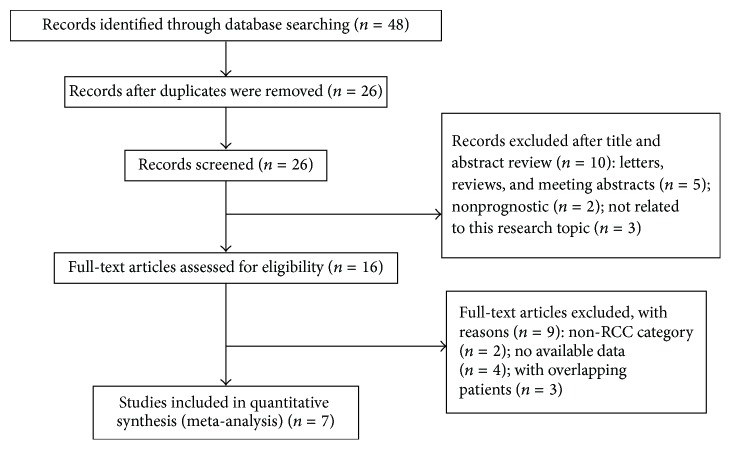
Flow chart of study selection.

The initial search identified 48 studies that were considered eligible according to the inclusion criteria. Eventually, 7 studies were included [10, 11, 15–19] (Figure 1). Two studies provided original information on the relationships between plasma fibrinogen and clinical parameters in RCC patients directly [10, 18]. The main characteristics of the 19 studies included in our meta-analysis are shown in Table 1. Our data has 3,744 patients from 6 countries (China, Austria, Turkey, Germany, Japan, and Korea).

Plasma fibrinogen levels were measured in 4 studies by a functional method based on the Clauss assay [11, 15–17]; fibrinogen tests were included in the coagulation panel among the preoperative workups in one study [19]; and, in the rest of the two studies, no comments were made on this point [10, 17]. Differences in the cut-off value for high plasma fibrinogen were observed among the studies. The high level of the plasma fibrinogen was considered to be positive, and a low level was considered to be negative.

### 3.2. Relationship between Plasma Fibrinogen and RCC Prognosis

**Figure 2:**
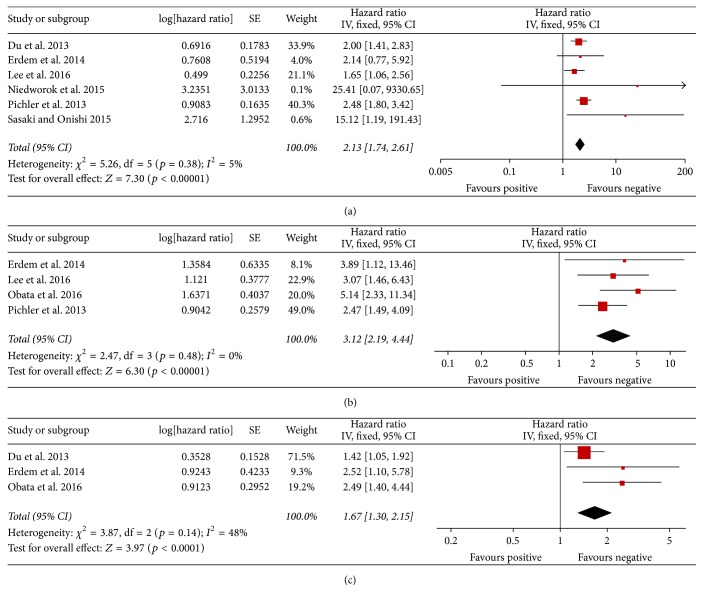
Results of subgroup analysis of the association between plasma fibrinogen and OS/CSS/DFS of RCC. (a) Six studies included investigating the relationship between OS and plasma fibrinogen. (b) Four studies included investigating the relationship between CSS and plasma fibrinogen. (c) Three studies included investigating the relationship between DFS and plasma fibrinogen. CI: confidence interval; CSS: cancer-specific survival; DFS: disease-free survival; OS: overall survival; RCC: renal cell carcinoma.

The forest plots of the meta-analyses for plasma fibrinogen are shown in Figure 2 and Table 2. The pooled HRs were statistically significant for OS (HR: 2.13; 95% CI: 1.74–2.61), CSS (HR: 3.12; 95% CI: 2.19–4.44), and DFS (HR: 1.67; 95% CI: 1.30–2.15).

### 3.3. Association between Plasma Fibrinogen in RCC and Clinical Parameters

**Figure 3:**
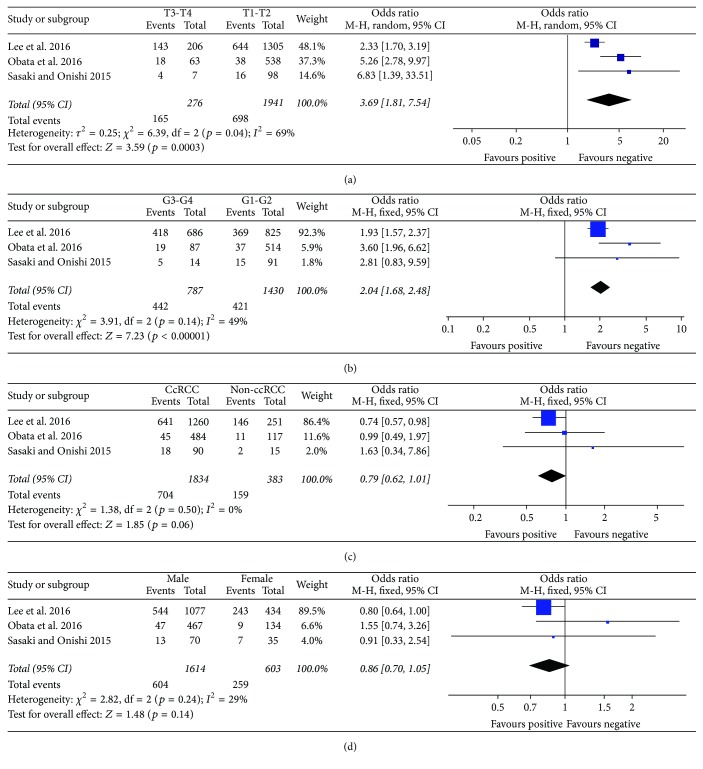
Results of subgroup analysis of the association between plasma fibrinogen and clinicopathological parameters. (a) The pooled OR from three studies including 1941 stage T1 and T2 and 276 stage T3 and T4 cases. (b) The pooled OR from three studies including 1430 grade G1 and G2 and 787 grade G3 and G4 cases. (c) The pooled OR from three studies including 1834 ccRCC and 383 non-ccRCC cases. (d) A total of 2277 RCC patients were pooled from three studies to assess whether plasma fibrinogen in RCC was associated with gender. ccRCC: clear cell renal cell carcinoma; RCC: renal cell carcinoma.

As shown in Figure 3(a), elevated plasma fibrinogen was significantly higher in advanced RCC (T3-T4) than in early stage RCC (T1-T2) (OR = 3.69, 95% CI: 1.81–7.54; *p* = 0.0003). The pooled OR from 3 studies including 1,430 RCC grade G1-G2 and 787 RCC grade G3-G4 patients is presented in Figure 3(b) (OR = 2.04, 95% CI: 1.68–2.48; *p* < 0.00001), which indicates that plasma fibrinogen was significantly higher in RCC patients of low Fuhrman grades than in those of high Fuhrman grades. The pooled OR from three studies, including 1834 ccRCC (clear cell renal cell carcinoma) and 383 non-ccRCC cases, is shown in Figure 3(c) (OR = 0.79, 95% CI: 0.62–1.01; *p* = 0.06), indicating that plasma fibrinogen was not strongly associated with tumor type in RCC patients. The pooled OR from four studies, including 1,601 males and 596 females, is shown in Figure 3(d) (OR = 0.86, 95% CI: 0.70–1.05; *p* = 0.14), indicating that plasma fibrinogen was not strongly associated with gender in RCC patients (Table 3).

### 3.4. Publication Bias

**Figure 4:**
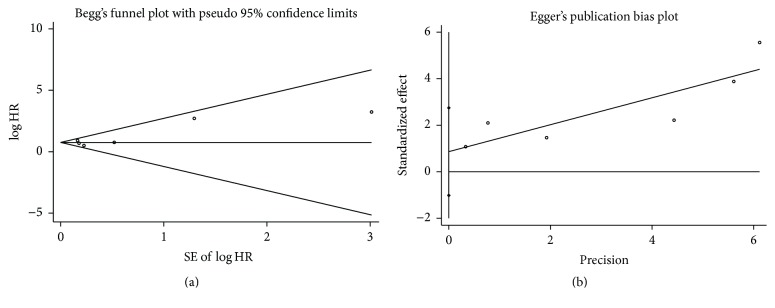
Funnel plots of Begg and Egger were used to detect publication bias on overall survival (OS). They showed no publication bias on OS in Begg's test (a) and Egger's test (b).

The Egger and Begg tests did not indicate any significant publication bias in the analysis of OS in RCC (*P*_begg_ = 0.707, *P*_egger_ = 0.272). No evidence of asymmetry was found in our funnel plot (Figure 4).

## 4. Discussion

Numerous researchers have reported various results relating plasma fibrinogen to RCC. However, up to now, no meta-analysis had been performed for the studies evaluating plasma fibrinogen as a prognostic marker in RCC.

In the current study, we enrolled 7 eligible studies comparing the correlations of RCC according to plasma fibrinogen. The individual data were organised according to OS, CSS, and DFS, and we identified the notion that an elevated plasma fibrinogen level predicts shorter OS, CSS, and DFS. Our results also indicate that RCC patients with elevated plasma fibrinogen level are likely to have a higher pathological T stage and a lower Fuhrman grade. The estimated pooled HRs of 7 trials for RCC were statistically significant, suggesting that plasma fibrinogen is a strong predictor of poor prognosis among patients with RCC. Our analysis helps to elucidate the results of individual studies which are related to the hypothesis that plasma fibrinogen is a prognostic factor for RCC, in addition to the identification of the high-risk subgroups of patients for whom adjuvant therapy may be useful.

The biological mechanism of plasma fibrinogen can explain its prognostic significance in RCC. It has been shown that tumor progression may set up a cascade of events which includes increased systemic inflammatory response, which in turn leads to increased plasma fibrinogen level [20–22].

Other studies show that fibrinogen can be endogenously synthesised by cancer cells [23, 24]. Fibrinogen is an extracellular matrix element and regulates the growth of cancer cells by binding to the vascular endothelial growth factor (VEGF), fibroblast growth factor-2 (FGF-2), and platelet-derived growth factor (PDGF) [24–26]. The binding of growth factors promotes cellular adhesion, proliferation, and metastasis during angiogenesis and tumor cell growth. Fibrinogen promotes platelets to adhere to tumor cells, and platelets also conversely induce more fibrinogen to aggregate around tumor cells by forming thrombin. Fibrinogen and platelets are promoted mutually and protect tumor cells from natural killer cytotoxicity [27]. Furthermore, using cell line models, it has been shown that highly concentrated fibrinogen can induce epithelial-mesenchymal transition (EMT) by increasing the expression of vimentin and reducing expression of E-cadherin, which enhances cancer cell invasion and metastasis [28]. Moreover, in vitro studies have shown that one possible mechanism is the association between tissue factor (TF) and VEGF. TF, which is expressed on the surface of tumor cells, is a key inducer of the coagulation pathway in carcinogenesis [29]. VEGF stimulates TF in endothelial cells, leading to activation of the coagulation cascade, which includes fibrinogen [25, 30]. Therefore, in RCC, which is characterised as a hypervascular tumor, it may be that an elevated plasma fibrinogen level is clearly associated with more aggressive pathological features and subsequent worse survival [16, 31].

To our knowledge, this meta-analysis is the first study to systematically evaluate the clinical and prognostic value of plasma fibrinogen level in RCC. The elevated plasma fibrinogen level predicted poorer pathological outcomes and was a significant risk factor affecting survival.

However, several limitations of this study need to be acknowledged. First, the applied methods for detecting plasma fibrinogen and the cut-off values were varied in the eligible studies, which could cause heterogeneity among the studies. Second, studies in other languages were excluded except for English; the literatures were not comprehensive. Third, other clinical factors such as race, age, and gender in each study might lead to bias. Fourth, subgroup analysis and metaregression were performed by type of RCC (clear cell RCC versus non-clear cell RCC); we lumped together the non-clear cell RCC group, but in this group there are a lot of different kinds of malignancies with different biological behaviors and genetic abnormalities, which might render the results less reliable. Finally, we could not ascertain a relationship between plasma fibrinogen and tumor type of RCC patients; clear cell RCC is more aggressive than other subtypes; however, only one study determined the plasma fibrinogen level differences between clear cell and other types and found no statistically significant differences. In this respect, other factors might also play a role in affecting RCC prognosis, such as clinical stage and Fuhrman grade.

In conclusion, this meta-analysis indicates that high plasma fibrinogen level is closely associated with poor survival and aggressive clinical feature in patients with RCC. While these are hypothesis generating results, the excellent accessibility and low cost of plasma fibrinogen should further facilitate its wider application in patients with RCC for risk stratification and decision-making of individualized treatment. We require further validation of our study.

## Figures and Tables

**Table 1:** Characteristics of individual studies included in the meta-analysis.

Study (year)	Country	Patients	Included period	Age (range) (year)	Gender (M/F)	Cut-off (mg/dL)	FU (range) (year)	Cofactors	NOS score
Du et al._2013	China	286	2000–2003	Median: 55.72 (28–77)	185/101	400	Median: 56 (34.6–94.5)	Hemoglobin, calcium, LDH, pT stage, Fuhrman grade, tumor size	7
Pichler et al._2013	Austria	994	2000–2010	Mean (63.2 ± 11.9)	599/395	466	Mean: 48.1 (0–132)	Age, gender, pT stage, Fuhrman grade, necrosis	8
Erdem et al._2014	Turkey	128	2006–2011	Mean (58.66 ± 11.31)	91/37	343	Median: 36.5	Gender, age, pT stage, Fuhrman grade, tumor size, histologic subtypes, plasma D-dimer	8
Niedworok et al._2015	Germany	98	2002–2011	Mean: 63.5 (18–82)	61/37	281	Mean: 36 (20–122)	NA	7
Sasaki and Onishi_2015	Japan	126	2003–2013	Median: 67 (37–86)	84/42	399	Median: 30.8 (2–125)	PS, pT stage, Hb, Alb, LDH	8
Obata et al._2016	Japan	601	1995–2010	Median: 58 (50–67)	467/134	420	Median: 74 (47–107)	Fuhrman grade, pT stage, histologic subtypes	8
Lee et al._2016	Korea	1511	2006–2013	Median: 58 (49–67)	1077/434	328	Median: 36 (24–57)	Age, BMI, hypertension, diabetes mellitus, ECOG score, tumor size, Fuhrman grade, pT stage, histologic subtypes, tumor necrosis, sarcomatoid differentiation	8

Alb: albumin; BMI: body mass index; ECOG: Eastern Cooperative Oncology Group; FU: follow-up; LDH: lactate dehydrogenase; Hb: hemoglobin; PS: performance status; NA: not available.

**Table 2:** HR values of the OS, CSS, and DFS of the RCC.

Outcome	Studies (*n*)	Patients	HR	95% CI	*p* value	Model	Chi^2^, *I* ^2^, *p* value
OS	6	3143	2.13	1.74–2.61	0.000	Fixed	5.26, 5%, 0.38
CSS	4	3234	3.12	2.19–4.44	0.000	Fixed	2.47, 0%, 0.48
DFS	3	1015	1.67	1.30–2.15	0.000	Fixed	3.87, 48%, 0.14

CI: confidence interval; CSS: cancer-specific survival; Fixed: fixed, inverse variance model; HR: hazard ratio; *I* ^2^: *I*-squared; OS: overall survival; Random: random, I–V heterogeneity model; DFS: disease-free survival.

**Table 3:** Plasma fibrinogen according to clinicopathological features.

Outcome of interest	Studies (*n*)	Patients	OR	95% CI	*p* value	Model	Chi^2^, *I* ^2^, *p* value
T3-T4 versus T1-T2	3	2217	3.69	1.81–7.54	0.0003	Random	6.39, 69%, 0.04
G3-G4 versus G1-G2	3	2217	2.04	1.68–2.48	0.000	Fixed	3.91, 49%, 0.14
CcRCC versus non-ccRCC	3	2217	0.79	0.62–1.01	0.06	Fixed	1.38, 0%, 0.06
Male versus female	3	2217	0.86	0.70–1.05	0.14	Fixed	2.83, 29%, 0.24

CcRCC: clear cell renal cell carcinoma; Fixed: fixed, inverse variance model; *I* ^2^: *I*-squared; OR: odds ratio; Random: random, I–V heterogeneity model; RCC: renal cell carcinoma.
